# Editorial Comment: Surgical Management of Peyronie's Disease With Co-Existent Erectile Dysfunction

**DOI:** 10.1590/S1677-5538.IBJU.2020.01.07

**Published:** 2020-01-13

**Authors:** P Krishnappa, E Fernandez-Pascual, J Carballido, I Moncada, E Lledo-Garcia, JI Martinez-Salamanca, Rodrigo R. Vieiralves

**Affiliations:** 1 Department of Urology, NU Hospitals, Bangalore, India;; 2 Department of Urology, Hospital Universitario Puerta de Hierro-Majadahonda, Madrid, Spain;; 3 Department of Urology and Robotic Surgery, Hospital La Zarzuela, c/Pléyades, Madrid, Spain;; 4 Department of Urology, Hospital General Universitario Gregorio Marañón, Madrid, Spain; 1 Serviço de Urologia, Hospital Federal da Lagoa, Rio de Janeiro, RJ, Brasil

Peyronie's disease (PD) is a connective tissue disorder with fibrotic plaque in the tunica albuginea, leading to penile deformity (1). For years, we sought to find a better form of treatment, from experimental studies in basic sciences to the present day with consolidated surgical modalities (2). As we know, a large number of patients have erectile dysfunction (ED) associated with Peyronie's disease. In most guidelines it is well established that penile prosthesis (PP) implantation is reserved for treatment of PD in patients with ED, especially when they are non-responders to oral therapy (3). In this scenario, the goal of PD surgery is to achieve a functionally straight penis (curvature <20 degrees) with good erection (4).

In this interesting paper, a systematic search on PubMed online database was done using the MeSH terms “Peyronie's disease” and “erectile dysfunction” with the objective to highlight the results of penile prosthesis to correct refractory erectile dysfunction in patients with PD.

One of the interesting aspects brought in the paper refers to the ideal moment of surgery. The authors observed that the ideal time is after three months of stable curvature or after 12 months from the onset of symptoms. Another important aspect is the type of prosthesis that should be used. The author provided data from a multicentric study involving 166 men evaluated on the significant difference in satisfaction and complication rates between the malleable penile prosthesis (MPP) and the Inflatable Penile Prosthesis (IPP) groups. Residual curvature was present in 6% of the patients with MPP and 16.7% with IPP with no statistical significance (5). According to the European Guidelines of Urology a small risk of urethral perforation (3%) has been reported in patients with ‘modeling’ over the inflated prosthesis (6). Although IPP is most preferred, there is no good level of evidence to prove that IPP is better than MPP. In regard to secondary procedures, such as manual modeling, plication, or graft placement, no new data was observed differing from the current guidelines. Grafts are usually preferred when penile curvature is more than 60 degrees. There is no ideal graft and the selection of a particular graft depends on the local availability, cost, and surgeon's expertise. At the end of the paper the authors propose an algorithm for the surgical correction of Peyronie's disease with coexisting erectile dysfunction. In this algorithm, after penile prosthesis implantation, manual modeling should be performed when residual curvature higher than 30° is observed. Tunica incisions or plication should be performed if persistent curvature after modeling is present. Plaque incision / excision and grafting should be done for persistent curvature (even after releasing incisions) or if defect longer than 2 cm is present.

This article presents a good overview of the surgical treatment of erectile dysfunction-related Peyronie's disease, covering the best indication of the various possible surgical alternatives. To conclude, we also emphasize that pre-operative counseling about the realistic outcomes of surgery in PD is mandatory to achieve adequate postoperative satisfaction rates.
